# An integrated overview of the immunosuppression features in the tumor microenvironment of pancreatic cancer

**DOI:** 10.3389/fimmu.2023.1258538

**Published:** 2023-09-12

**Authors:** Jinglong Guo, Siyue Wang, Qi Gao

**Affiliations:** ^1^ Department of Cardiovascular Disease, the First Hospital of Jilin University, Changchun, China; ^2^ Baylor College of Medicine, One Baylor Plaza, Houston, TX, United States

**Keywords:** pancreatic cancer, complex tumor microenvironment, diverse cellular cross-regulations, immunosuppression features, targeting strategy

## Abstract

Pancreatic ductal adenocarcinoma (PDAC) is one of the deadliest malignancies. It is characterized by a complex and immunosuppressive tumor microenvironment (TME), which is primarily composed of tumor cells, stromal cells, immune cells, and acellular components. The cross-interactions and -regulations among various cell types in the TME have been recognized to profoundly shape the immunosuppression features that meaningfully affect PDAC biology and treatment outcomes. In this review, we first summarize five cellular composition modules by integrating the cellular (sub)types, phenotypes, and functions in PDAC TME. Then we discuss an integrated overview of the cross-module regulations as a determinant of the immunosuppressive TME in PDAC. We also briefly highlight TME-targeted strategies that potentially improve PDAC therapy.

## 1 Introduction

Pancreatic cancer is the third leading cause of cancer-related death in Western countries (1). Concerningly, it has been estimated there would be 64,050 people diagnosed with pancreatic cancer and 50,550 people would die from it in 2023 in the United States (1). This disease is mostly diagnosed at advanced stages, making current therapeutic regimens rather ineffective (2, 3). Pancreatic cancer is rapidly lethal, with an overall 5-year survival rate of only 11% (2, 3). Surgical resection and adjuvant chemoradiotherapy are viable options for only 10-20% of newly diagnosed patients, resulting in a 5-year survival rate of 15-25% among this subgroup (2, 3). Currently, most patients with advanced pancreatic cancer are mainly treated with chemotherapy regimens such as FOLFIRINOX (i.e., combination of drugs leucovorin calcium (folinic acid), fluorouracil, irinotecan hydrochloride, and oxaliplatin) and gemcitabine/nab-paclitaxel, however, their overall efficacy remains significantly limited, with the median overall survival < 1 year (2, 3). The factors causing the lethality of pancreatic cancer are multifaceted, including multiple germline mutations, poor diagnosis, resistance to conventional therapies, and highly immunosuppressive tumor microenvironment (TME) (2–5).

Pancreatic ductal adenocarcinoma (PDAC) is the most common type of pancreatic malignancy (greater than 90%) (2). It features a complex TME that is composed of diverse acellular and cellular components, mostly including dense extracellular matrix (ECM), tumor cells, stromal cells, and immune cells (4, 5). Interactions between these various cellular elements occur through direct cell-cell contact and indirect communication mediated by secreted molecules, culminating in the establishment of an immunosuppressive milieu (4, 5). The immunosuppression feature has been recognized as a general hallmark of PDAC TME, characterized by heightened infiltration of tumor-promoting myeloid cells including tumor-associated macrophages (TAMs), tumor-associated neutrophils (TANs), myeloid-derived suppressor cells (MDSCs), and mast cells, along with impaired number and function of anti-tumor immune cells such as CD8 T cells, Dendritic cells (DCs), and natural killer cells (NKs) (4, 5). Concomitantly, this immunosuppressive milieu substantially influences the development, prognosis, and treatment outcomes of PDAC (4, 5).

Immunotherapies, such as immune checkpoint inhibitors (ICI) that disrupt the inhibitory pathways of T cells and thereby unleash their power against cancer, have revolutionized treatment paradigms for a range of human cancers over the past decade (2, 3, 6). However, PDAC has been reported to extremely resist monotherapy with ICIs (2, 3, 6), which likely attributes to the highly immunosuppressive nature of the PDAC TME (4, 5, 7). In this regard, we argue that an integrated understanding of the immunosuppressive TME will open new targeted opportunities to improve PDAC therapy more effectively. In this review, we integrate cellular sub(types), phenotypes, and functions of the diverse cellular components in PDAC TME to summarize five cellular composition modules. Then we discuss a comprehensive overview of the cross-module interactions and regulations as a potent determinant of the immunosuppressive TME in PDAC. Lastly, we briefly highlight novel TME-targeted approaches that potentiate the improvement of PDAC therapy.

## 2 Overview of five cellular composition modules in PDAC TME

**Figure 1:**
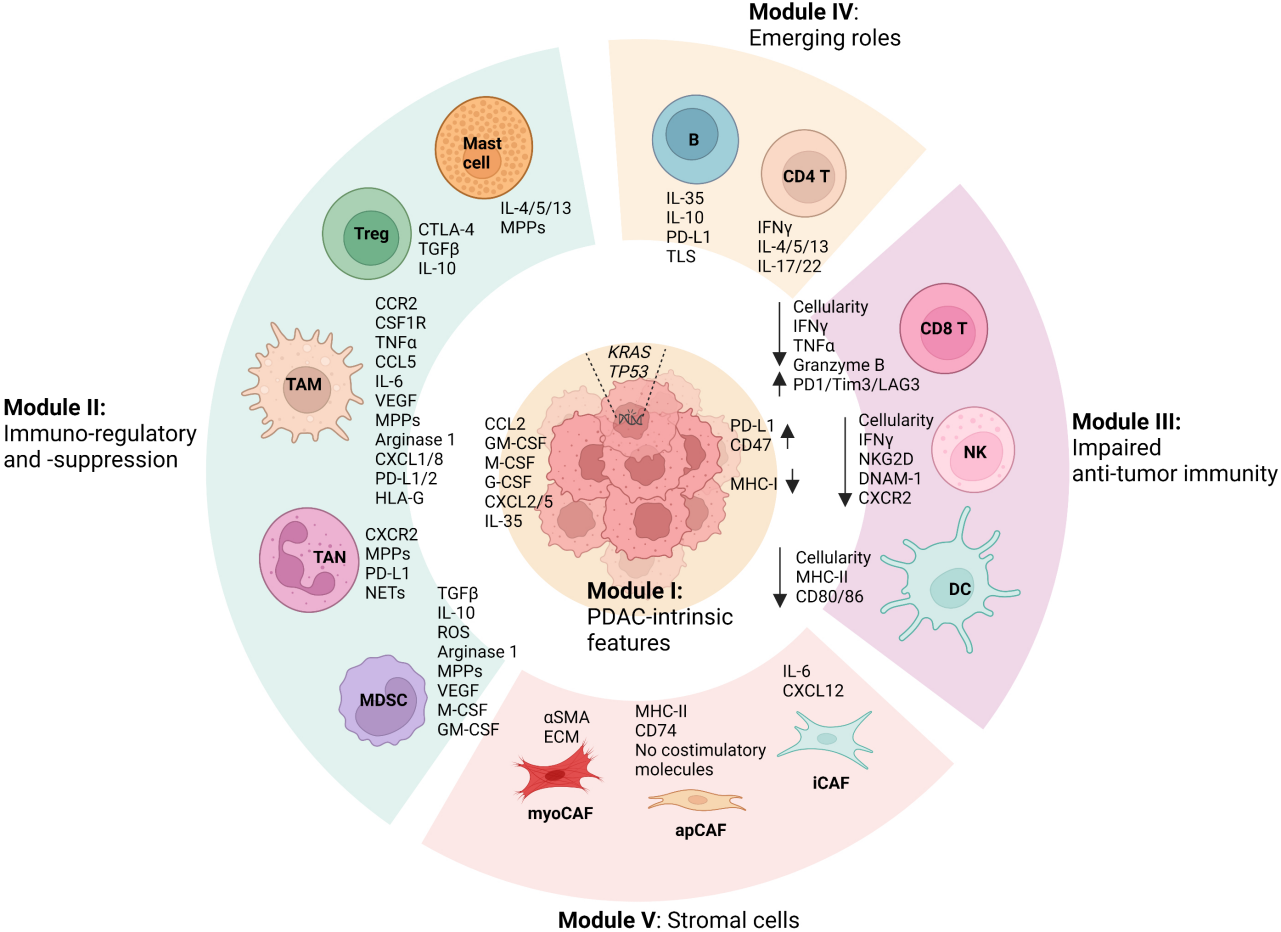
Summary of modular cell compositions and molecule mediators in PDAC TME. Pancreatic ductal adenocarcinoma (PDAC)-intrinsic features such as genetic mutations and related signal pathways are concluded as Cellular Module I, as they have been reported to profoundly shape the formation of the immunosuppressive tumor microenvironment (TME). Immunoregulatory and immunosuppressive Module II is primarily composed of tumor-associated macrophages (TAMs), tumor-associated neutrophils (TANs), myeloid-derived suppressor cells (MDSCs), mast cells, and regulatory T cells (Tregs), which play significant roles in fostering PDAC progression and in suppressing anti-tumor immunity. In PDAC TME, anti-tumor immune cells including CD8 T cells, Dendritic cells (DCs), and natural killer cells (NKs) are profoundly impaired in terms of both number and function, which can be dramatically regulated by the cells from other cellular modules, especially Module II. Besides, CD4 helper T cell subsets (Th1, Th2, and Th17) and B cells in PDAC TME have been shown to play either tumor-suppressing or tumor-supporting roles in context-dependent manners, of which emerging roles are discussed in Module IV. Lastly, the heterogeneity and functions of cancer-associated fibroblasts (CAFs) in the context of immunosuppressive TME of PDAC is summarized as Stromal module V, which includes myofibroblast-like CAFs (myoCAFs), inflammatory CAFs (iCAFs), and antigen-presenting CAFs (apCAFs). In addition, the primary molecular mediators used by the cells in terms of their functions discussed in the review are shown accordingly. Remarkable cross-interactions and -regulations among cellular modules occur through the molecular mediators, culminating in the formation of an immunosuppressive TME that essentially influences PDAC oncological hallmarks and treatment efficacy. (The figure was created in Biorender with the publication license).

PDAC exhibits high genetic heterogeneity and is characterized by an overarching TME, where diverse cellular compositions and acellular mediators contribute to a remarkable desmoplastic reaction (4, 5). Recent evidence has established the notion that the TME of PDAC is dominated by immunosuppression features, which significantly influence PDAC phenotypes and treatment outcomes including both conventional chemotherapies and revolutionary immunotherapies (4, 5). A comprehensive understating of the diversity and interactions within PDAC TME that unravels the mechanistic determinant of its immunosuppression feature will shed light on the development of new therapeutic interventions (4, 5). To this end, we integrate the cellular (sub)types, phenotypes, and functions of the diverse cellular components within PDAC TME and summarize five cellular composition modules (**Figure 1**). First, PDAC-intrinsic aspects are concluded as (I) the Tumor cell module since PDAC genetic mutations and related signal pathways have been recognized as a critical factor driving the formation of the immunosuppressive TME (8–11). (II) The Immunosuppression module is mostly composed of TAMs, TANs, MDSCs, Treg cells, and mast cells, given that they constitute an abundant component in PDAC TME and play notorious immunoregulatory and immunosuppressive roles (12–14). Particularly, its immunosuppressive capacity is significantly overwhelming compared with the anti-tumor immunity including CD8 T cells, DCs, and NKs, which are impaired in number and function in PDAC TME and therefore drive us to define (III) the Anti-tumor immunity impaired module (15–18). Besides, immune cells including CD4 helper T cell subsets (Th1, Th2, and Th17) and B cells have been shown to display features of a double-edged sword in PDAC TME and play either tumor-suppressing or tumor-supporting roles in context-dependent manners. Thus, emerging roles for them in PDAC TME and cancer immunity are discussed accordingly in the IV Module (19–23). Lastly, we describe the heterogeneity and functions of cancer-associated fibroblasts (CAFs) in the context of immunosuppressive TME of PDAC in (V) the Stromal module (24). By the summary and explicit discussion (in the following paragraphs) of above five cellular composition modules in PDAC TME, we argue that there are cross-interactions and -regulations among cellular modules that represent a resultant force essentially dictating the immunosuppression features, PDAC oncological hallmarks, and treatment efficacy. It is worth noting that each cell population in PDAC TME may exhibit a high degree of plasticity, and their behavior may not strictly align with the originally defined modules, particularly in the context of therapeutic interventions. Therefore, understanding and accounting for this plasticity is vital for developing effective PDAC treatment strategies.

## 3 PDAC-intrinsic aspects: the primary driver of immunosuppressive TME formation

An expanding body of evidence from preclinical mouse model studies and clinical observations underscores the crucial role of genetic mutations in oncogenes and/or tumor-suppressor genes in shaping important PDAC features, including tumor cell differentiation and heterogeneity, histopathological subtypes, and clinical outcomes (8–11, 25, 26). Besides, defined oncogenic mutations are also associated with changes in the composition of immune cells and immunotherapy efficacy in PDAC (8–11, 25, 26). A comprehensive understanding of how genetic oncogenes and related signaling pathways affect PDAC phenotypes and immune cell composition and function will provide valuable insights for the development of precise targeted therapies and immunotherapies aimed at combating this aggressive malignancy.

### 3.1 Genetic mutations drive PDAC initiation and progression

PDAC progresses from noninvasive precursor lesions, including pancreatic intraepithelial neoplasia (PanIN), intraductal papillary mucinous neoplasm (IPMN), intraductal tubulopapillary neoplasm (ITPN), and mucinous cystic neoplasm (MCN) (26). Among them, PanINs are the most well-characterized preneoplasia lesions so far (26). PanINs originate within intralobular ducts and can be further classified into four grades, PanIN 1A, PanIN 1B, PanIN 2, and PanIN 3 (26). Of note, all preneoplastic lesions are likely to reflect the PDAC progression. Genetic mutations are the primary driver of PDAC initiation and progression (11, 25). It has been reported that a PDAC patient usually harbors 32 genetic mutations on average (11, 25). Among the mutations, activating mutations in KRAS are present in over 90% of tumors (11, 25). The mutations of cell cycle checkpoint genes, like CDKN2A, TP53, and SMAD4 account for 50-80% of cases (11, 25). In addition to these common mutations, there are less frequent mutated genes (-10% of tumors), including ARID1A, MLL3, and TGFBR2, which can contribute to a more aggressive phenotype of PDAC (11, 25). Despite enduring research endeavors, targeted therapies have not yet demonstrated significant benefits for PDAC patients (27).

### 3.2 Genetic mutations in PDAC cells shape immunosuppressive TME formation

Emerging evidence has shown that oncogenic mutations in cancer cells primarily dictate the immune contexture in the TME (9). Deciphering the underlying relationship between cancer cell-intrinsic genetic events and the immune cell contexture in the TME may enable the improvement of both chemotherapies and immunotherapies for cancer patients. We highlight a few examples of the studies to discuss how indicated oncogenic mutations in cancer cells modulate the immune cell composition and function in the TME of PDAC.

Oncogenic K-RAS represents one of the most abundant and common mutations during PDAC initiation and progression (11, 25). K-RAS mutations are involved in several signaling pathways such as RAF/MEK/ERK and PI3K/AKT/mTOR (28, 29). They not only determine PDAC phenotypes but also significantly regulate the immunosuppressive TME (28, 29). For example, studies from Pylayeva-Gupta et al. and Bayne et al. independently reported that oncogenic K-RASG12D in mouse pancreatic ductal epithelial cells drove elevated GM-CSF secretion, thereby recruiting Gr1+CD11b+ MDSCs into PDAC TME (30, 31). Further studies showed that neutralizing GM-CSF genetically or pharmacologically in mice was sufficient to reduce these cells, along with elevated tumoral infiltration of CD8 T cells and slowed PDAC growth (30, 31). It thus suggests GM-CSF and/or MDSCs to be potential targets for PDAC therapy. Additionally, K-RAS mutations are also involved in the suppression of innate and adaptive anti-tumor immunity through modulating PDAC expressions of immune checkpoints such as PD-L1 and CD47 (32, 33), as well as through autophagy-mediated MHC-I downregulation in PDAC (34, 35).

As one of the molecule events downstream of RAS signaling pathways, MYC activation and overexpression are commonly found in PDAC (36, 37). Beyond regulating PDAC phenotypes, MYC has also been linked to the immunosuppressive TME (38, 39). Using mouse models of PDAC that carry K-RASG12D and inducible MYC-ERT2, Sodir et al. showed that acute activation of MYC triggered rapid changes in stromal and immune cells (38). This included a marked influx of F4/80+CD206+ TAMs and Ly-6B.2+ neutrophils, significant loss of B220+ B lymphocytes and CD3+ T cells, and induction of α-SMA in proximal stellate and fibroblastic cells (38). As a result, it established a TME reminiscent of human PDAC (38). Interestingly, subsequent MYC deactivation or inhibition immediately reversed the advanced PDAC phenotypes back to PanIN, suggesting the requirement of sufficient levels of MYC for instructing the PDAC phenotypes and TME features (38). In this regard, elevated levels of MYC in tumor cells have been shown to promote PDAC metastasis through CXCL13- and macrophage migration inhibitory factor (MIF)-mediated recruitment of TAMs in a recent study (39). Additionally, concomitant MYC and K-RASG12D expression caused suppression of Type I IFNs, thereby resulting in decreased NK and B cell infiltration and advanced PDAC phenotypes (40). Together, these studies suggest an important role for MYC in dictating the immunosuppressive TME of PDAC and provide compelling insights for therapeutically targeting MYC.

The tumor suppressor TP53 mutations occur in 50-70% of human PDAC, which have been shown to affect immune cell composition in PDAC TME (8, 41). By analyzing human PDAC patient data from The Cancer Genome Atlas (TCGA), Maddalena et al. reported the significant association of TP53 missense mutations with reduced frequency of CD8 T cells in human PDAC (41). In addition, using mouse models of PDAC carrying p53R172H mutation, Siolas et al. reported an elevated secretion of CXCL2 and CXCL5, thereby leading to the accumulation of CD11b+Ly6G+ neutrophils in TME (42). On the other hand, p53 loss in mouse PDAC cells caused decrease of CD4 and CD8 T cells whereas increase in immunosuppressive CD11b+ myeloid cells and Treg cells in PDAC TME (42, 43). Thus, these data demonstrate a contribution of TP53 mutations to the immunosuppressive TME of PDAC.

## 4 Immunoregulatory and immunosuppressive cells: the main executor of immunosuppression in PDAC TME

### 4.1 Tumor-associated macrophages

Tumor-associated macrophages (TAMs) are abundant in the TME of PDAC. These cells appear to play important but potentially various roles in fostering tumorigenesis, shaping the TME, and suppressing anti-tumor immunity (44, 45). TAMs promote PDAC initiation and progression by secreting a variety of proinflammatory cytokines including TNFα, RANTES (CCL5), and IL-6 (46, 47). For instance, it has been reported that TAMs-secreted TNFα and RANTES activated NF-κB in acinar cells to drive their proliferation and survival. In turn, acinar cells expressed intercellular adhesion molecule-1 (ICAM-1) to mediate their cellular adhesion with TAMs. Thus, TAMs and acinar cells formed a paracrine loop, sustaining local inflammation and inducing acinar-to-ductal metaplasia (ADM) transformation in the early stage of carcinogenesis (46, 47). IL-6 can contribute to the development of the early premalignant pancreatic lesions ADM and PanIN through JAK-Stat3 or Stat3/Socs3 pathways (48, 49). Moreover, TAMs can regulate tumoral neoangiogenesis, epithelial-mesenchymal transition (EMT), and PDAC metastasis (44, 45). In response to TME hypoxia, TAMs upregulate the expression of HIF-1α, a master transcriptional factor that regulates cellular and tissue adaptive responses to hypoxia (44, 50). HIF-1α further regulates the expression of numerous angiogenesis-related genes such as VEGF, PDGF, βFGF, IL-1β, IL-8, TNF-α, thymidine phosphorylase, MMPs, CXCL1, and CXCL8 (44, 50). For example, by depleting TAMs pharmacologically or genetically in mouse models of PDAC, Griesmann H demonstrated a significant reduction in liver metastasis of tumor cells and impairment of neoangiogenesis. In addition, the study showed the presence of VEGF-expressing TAMs in pre-metastatic niches and their depletion caused the decrease in circulating VEGF levels. Based on these data, the authors claimed that VEGF-expressing TAMs promoted tumor cell extravasation and vascular permeability (51). Of note, the studies have not provided a clear answer regarding whether the observed effects were directly mediated by VEGF or influenced by other factors. Furthermore, functioning as immunosuppressive cells, TAMs produce a variety of immunoregulatory cytokines such as TGFβ, IL-10, and prostaglandin E2 (PGE2) and express inhibitory molecules PD-L1 and PD-L2, which promote Treg cell infiltration and inhibit anti-tumor CD8 T cell activity (44, 45). Besides, TAMs also suppress NK cell function by upregulating the expression of HLA-G, an inhibitory molecule for NK cells (44, 52). In summary, TAMs promote PDAC initiation and progression, regulate TME immunosuppression, and inhibit anti-tumor immunity. Nonetheless, TAMs are heterogenous and high of plasticity, therefore possessing significant potential to mediate anti-tumor responses when purposely modulated.

Historically, TAMs have been recognized to exclusively originate from the differentiation of recruited monocytes (MoMΦ) (44, 45). However, recent studies demonstrated that they also derived from the expansion of pancreatic tissue-resident macrophages (TRM) in PDAC, which were phenotypically and functionally distinct from MoMΦ (53). In mouse models, CCR2 genetic knockout mediated-MoMΦ selective depletion didn’t affect PDAC growth, indicating a dispensable role for them in tumor progression (53). Interestingly, PDAC growth was remarkably reduced in mouse models with specific depletion of pancreatic TRMs (53). These data suggested that TRM expansion-derived TAMs were more robust drivers of PDAC progression than MoMΦ (53). It is worth mentioning that macrophage heterogeneity has long been defined as M1 and M2 macrophages based on *in vitro* polarization studies (44, 45). Briefly, M1 macrophages are classically induced by bacterial products (lipopolysaccharide) and/or pro-inflammatory cytokines (IFNγ and TNFα), produce proinflammatory cytokines (such as IL-12, CXCL10), and mediate protective immune responses. By contrast, M2 macrophages are alternatively activated by immunoregulatory cytokines (such as IL-4, IL-10, or IL-13), producing factors (such as VEGF) associated with wound healing and tissue repair (44, 45). Of Note, more and more evidence has argued that TAMs rarely express bona-fide M1 or M2 phenotypes, implying that the diversity of these cells cannot simply be addressed with this binary categorization.

Recently, one of the striking research advancements in the field has been the characterization of the TAMs that are positive for triggering receptor expressed on myeloid cells 2 (TREM2) (54–56). TREM2 is overexpressed on TAMs in 75% of human tumors and its expression highly correlates with poor tumor prognosis in patients (54). Studies conducted on mice reveal that TREM2+ macrophages dampen the anti-tumor activities of CD8+ T cells and NK cells, signifying bona-fide immunosuppressive functions for these cells (54–56). Moreover, TREM2 modulation by genetic ablation or monoclonal antibodies can remodel the myeloid cell immunosuppression within the TME, restrict tumor growth, and further improve immunotherapies such as anti-PD-1 therapy and NK cell-based therapy in mouse models with different tumor types (54–56). Notably, fundamental questions regarding how TREM2 expression is induced in TAMs and how TREM2 regulates the immunosuppressive phenotypes of TAMs remain largely elusive (57). Potential explanations could involve in the TAM metabolism, given that TREM2 is a receptor for a wide array of ligands, including anionic molecules, DNA, lipoproteins, and phospholipids. These ligands are intimately associated with cellular metabolism and are abundantly present in the TME (57).

### 4.2 Tumor-associated neutrophils

Neutrophils represent one of the most abundant leukocytes in the blood of humans (up to 50-70%) in physiological settings, which have drawn a lot of attention to investigate their relationship with cancer (58). There were studies to report that PDAC cells, even tumor cells from the lesions of early stages such as PanIN, can significantly promote the expansion of neutrophil progenitors in bone marrow by secreting growth factors (GM-CSF, G-CSF, and M-CSF) (59, 60). Accordingly, the Neutrophils-Lymphocyte Ratio (NLR) of periphery blood has been identified as a faithful prognostic value of the outcomes of PDAC patients after treatment (61, 62). Specifically, a high NLR value (NLR>2.5) was remarkably associated with a decreased frequency of CD8 T cells within the tumor and predicted worse overall survival in PDAC patients after surgical resection and chemotherapy (57, 58). More recently, by analyzing PDAC mouse models and PDAC samples of patients, Jiang et al. found that neutrophil infiltration displayed a body-wide effect, including liver, lung, colon, stomach, kidney, heart, and brain (63). Thus, systemic neutrophil infiltration and associated inflammation can be a cautious marker of pancreatic cancer prognosis.

In addition to promoting neutrophil progenitor expansion in bone marrow, PDAC is involved in recruiting neutrophils to the TME and pre-metastatic niches through secretion of a variety of chemokines such as CXCL1, CXCL2, CXCL5, and CXCL8 (64–67). Using mouse models, Steele et al. reported the liver recruitment of CXCR2+ neutrophils contributing to PDAC metastasis (67). In the context of CXCR2+ neutrophil depletion genetically or pharmacologically, PDAC liver metastasis was remarkably reduced, along with significantly prolonged tumor-free survival of PDAC mouse models (67). Although not directly investigated in the study, it is reasonable to propose a link between the mechanistic action of CXCR2+ neutrophils in PDAC and CXCR2 ligands, given that CXCR2 is a receptor for a series of chemokines CXCL1, CXCL2, CXCL3, CXCL5, CXCL6, CXCL7, and CXCL8 (66, 67). Furthermore, CXCR2+ neutrophil depletion improved tumoral infiltration and function of CD8 T cells, which sensitized anti-PD1 therapy in mouse models of PDAC (67). Together with reports showing that TANs expressed PD-L1 to suppress anti-tumor T cell functions, TANs therefore have been considered as significant immunosuppressive cells in PDAC TME (66–68). Besides, like TAMs, TANs in PDAC TME are also a substantial source of ECM degradation mediators such as MMPs and Elastase, which can promote PanIN progression, PDAC invasiveness, and metastasis (58, 69). In recent years, the role of neutrophil extracellular traps (NETs) has gained attention in neutrophil biology and related diseases (70). NETs are network structures composed of DNA-histone complexes and proteins released by activated neutrophils (70). Studies showed that NETs activated IL-1β/EGFR/ERK pathway, and subsequently promoted PDAC EMT and metastasis (71, 72). Collectively, these studies support the notation that TANs represent one of the major immunosuppressive populations in the TME, inhibiting anti-tumor immunity and contributing to PDAC progression.

Like the definition of M1 and M2 for macrophages, TANs have been classified as N1 (anti-tumor) and N2 (pro-tumor) based on the activation and functional status in the TME (73). Fridlender et al. showed that TGFβ-induced differentiation of N2 TANs led to a pro-tumor phenotype in TME, whereas anti-tumor N1 TANs were polarized when TGFβ was ablated. The study highlighted the phenotypic and functional heterogeneity of TANs in PDAC TME (73). Another important question is what are the functions of TANs in the context of cancer immunotherapy? Recent studies have shown that immunotherapy-activated T cells can recruit and induce the maturation of neutrophils, leading to an improved capacity of neutrophils to directly kill tumor cells (74, 75). This demonstrates an important role for neutrophils in the context of cancer immunotherapy.

### 4.3 Myeloid-derived suppressor cells

Myeloid-derived suppressor cells (MDSCs) are a heterogeneous population originating from myeloid progenitor cells of bone marrow. They are primarily classified into two populations, mononuclear (M-MDSC) and polymorphonuclear cells (PMN-MDSC), which accounts for 20-30% and 70-80% of the total MDSC population, respectively, in most tumors (76–78). Both M-MDSCs and PMN-MDSCs were reported to remarkably accumulate and expand in PDAC TME, and importantly, their frequency was negatively correlated with patient survival and response rates of immunotherapies (76, 77). M-MDSCs express higher levels of signal transducer and activator of transcription 1 (STAT1), inducible nitric oxide synthase (iNOS), and nitric oxide (NO, which is produced by iNOS-mediated L-arginine metabolism) (78). On the other hand, PMN-MDSCs have increased levels of STAT3, reactive oxygen species (ROS), but less NO (78). ROS is a detrimental agent for T cells, while simultaneously maintaining the survival of MDSCs themselves (78). These cellular events result in the suppression of anti-tumor T cell responses (78). Importantly, both M-MDSCs and PMN-MDSCs are an important source of arginase 1, which deprives L-arginine required for T cell metabolism, thus impairing their functions (76, 78). In PDAC TME, MDSCs (including both M-MDSCs and PMN-MDSCs) also directly maintain other immunosuppressive cells including TAMs and Tregs (76, 79). For example, by using light sheet fluorescent microscopy, Siret et al. observed the close associations of MDSCs and Tregs in tumor samples from PDAC mouse models and patients. Further studies demonstrated that MDSCs secreted TGFβ and IL-10, fostering Treg frequency and functions locally in PDAC TME (79). Besides, like TAMs and TANs, MDSCs can also promote neoangiogenesis, EMT, and metastasis of PDAC through secretion of a variety of mediators such as G-CSF, GM-CSF, stem cell factor (SCF), cyclooxygenase 2 (COX-2), PGE2, MPPs, VEGF, and HGF (78, 80). Lastly, it is especially worth mentioning that PMN-MDSCs are distinct from neutrophils, given that they have increased levels of arginase 1 and peroxynitrite, fewer granules, and reduced CD16 and CD62L expression (78, 80).

### 4.4 Regulatory T cells

Regulatory T cells (Tregs) are a subset of immunosuppressive cells, which have been largely reported to play tumor-promoting roles (81). Tregs are highly infiltrated into PDAC, and their abundance is often correlated with a poor prognosis and reduced survival in patients (82). Tregs exert their immunosuppressive effects through various mechanisms. One of the main mechanisms employed by Tregs is the expression of immune inhibitory molecules, such as CTLA-4, which can dampen the activation and function of CD8 T cells (81). Additionally, Tregs produce immune regulatory cytokines, including TGFβ and IL-10, which further contribute to the suppression of anti-tumor immune responses. Moreover, they also compete with other T cells for IL-2 via higher expression of the IL-2 receptor, and therefore suppressing T cell function (81). Interestingly, a recent study showed that depletion of Tregs accelerated PDAC growth due to compensatory infiltration of tumor-promoting myeloid cells, specifically, TAMs (83). The specific mechanisms underlying this phenomenon were not explored in the study, highlighting the need for further research. Nevertheless, these findings suggest that caution should be exercised when considering Treg depletion as a therapeutic strategy for PDAC.

### 4.5 Mast cells

Mast cells are also one of the immune cell subsets that have been shown increased infiltration in PDAC. There were studies to report the inverse correlations between the frequency of mast cells with pathological grades of tumors and the overall survival of patients with PDAC (84, 85). Chang et al. observed the increased infiltration of mast cells into the tumors in a mouse model of PDAC, compared to that in the pancreas of healthy mice (85). Furthermore, they orthotopically transplanted the PDAC cells, that were isolated from the mouse model, into mast cell-deficient mice (Kit^w-sh/w-sh^) and found that the tumor growth was significantly slower than that in WT recipient mice. Reconstitution with mast cells in Kit^w-sh/w-sh^ mice remarkably restored PDAC growth. These studies thus demonstrated a tumor-promoting role for mast cells in PDAC growth (85). In fact, mast cells have been shown to secrete a variety of tumor-supporting and/or immunoregulatory factors in PDAC TME, including IL-13, Tryptase, MMPs (84, 86). Despite the evidence pointing towards a tumor-promoting role for mast cells in PDAC, the specific targeting strategies for mast cells in PDAC therapies have received limited investigation so far.

## 5 Impaired anti-tumor immunity in PDAC TME: CD8 T cells, DCs, and NK cells

### 5.1 CD8 T cells

Cytotoxic CD8 T cells play a central role in anti-tumor immunity. Upon recognition of T cell receptor (TCR) of tumor cells, of which tumor-specific antigen is presented by major histocompatibility complex class I (MHC-I), CD8 T cells can kill tumor cells through producing cytotoxic molecules, such as granzymes and perforin (15). Generally, high tumoral infiltration of CD8 T cells and/or their improved functional status are positively associated with responses to therapies and the outcomes in patients across many cancer indications (15, 87). In most patients with PDAC, however, CD8 T cells are either scarce or excluded from the tumor cores. Even though CD8 T cells are present intratumorally in some PDAC patients, they are usually dysfunctional or exhausted, evidenced by elevated expression of a set of checkpoint molecules including PD-1, Tim-3, and LAG-3, and reduced production of effector cytokines such as IFNγ, TNFα, and granzyme B. Many mechanisms that mediate PDAC immune escape have been reported (15, 87). For example, Yamamoto et al. showed that autophagy-mediated degradation in PDAC contributed to significantly downregulated MHC-I molecules, consequently preventing CD8 T cells from being fully activated (34). In general, it appears that nearly all cell types from the defined Immunosuppression module, including TAMs, TANs, MDSCs, and Treg cells, can suppress CD8 T cells (**Figure 1**) (13, 14). CAFs also contribute to CD8 T cell suppression through the secretion of immunoregulatory molecules such as TGFβ and CXCL12 (88, 89), as well as through forming a physical barrier to directly prevent their infiltration (90). In summary, CD8 T cells in the tumors of most PDAC patients are rare, dysfunctional, and excluded from the tumor cores.

### 5.2 Dendritic cells

Dendritic cells (DCs) are professional antigen-presenting cells and initiate immune responses when fully activated. Numerous studies have reported that DC numbers and functions are significantly low in PDAC samples of patients, compared with other tumor types (91, 92). Accordingly, most PDAC patients were found the remarkable lack of circulating DCs in peripheral blood, who were usually associated with worse survival outcomes. It suggested the significance of DCs in PDAC patients (91, 92). In this regard, immense efforts have been put into the development of DC-based therapies for PDAC (16, 93). For example, using tumor antigen-expressing mouse models of PDAC and lung cancer, Hegde et al. reported a remarked impairment of conventional DCs in numbers and functions in PDAC, but not in lung tumors, which resulted in different tumor controls (16). It was further demonstrated that treatment with Flt3L and CD40 agonism, a regimen to improve DCs, led to PDAC control. Importantly, this treatment rendered PDAC responses to radiotherapy and its control was further improved (16, 93). These studies suggest a significant potential of DC-based therapies for PDAC. However, the reasons causing the impairment of DCs in PDAC remain to be further characterized.

### 5.3 Natural killer cells

Natural killer cells (NK cells), a population of innate lymphoid cells, are important players in the immune surveillance of cancer. NK cell activation is controlled by integrating signals from activation and inhibitory receptors. Normal cells express MHC-I molecules, the ligands for the inhibitory receptors of NK cells, to keep them under check. On the other hand, tumor cells usually downregulate MHC-I to escape from CD8 T cell killing, making them susceptible to NK cell-mediated killing. Hence, NK cells and CD8 T cells coordinate to keep effective immune surveillance of tumor cells. However, both NK cells and CD8 T cells (abovementioned) are impaired in PDAC TME (18, 94). Lim et al. provided evidence showing a lower frequency of NK cells in tumor samples of PDAC patients, which was due to downregulated expression of CXCR2, a receptor of several chemokines important for NK cell recruiting (94). Furthermore, NK cell functional impairment was also evidenced, mechanistically attributed to decreased expression of NK cell activation receptors NKG2D and DNAM-1 (94). The molecular insights leading to the NK cell impairment in PDAC TME, such as what causes downregulated CXCR2, NKG2D, and DNAM-1 in NK cells, remain largely unknown. More recently, Muthalagu et al. provided a novel mechanistic study to explain the NK cell evasion in PDAC (40). Using mouse models of PDAC expressing oncogenes MYC and K-RAS, they showed that type I IFNs were suppressed due to the binding of repressive MYC-MIZ1 complexes directly to the gene promoters of type I IFN regulators IRF5, IRF7, STAT1, and STAT2. Consequently, it contributed to the ineffectiveness of NK cell infiltration and PDAC control. Further study showed that genetic or pharmacological removal of repressors of type I IFN regulator genes increased NK cell infiltration and mouse survival. This study not only shed light on the mechanisms underlying NK cell impairment but also highlighted the possibility of targeting IFN signaling to improve PDAC therapy (40). In addition, NK cell cytotoxicity and INFγ production can be impaired by TGFβ, an abundant cytokine of immunoregulatory in PDAC TME (18, 95). Therefore, strategies to restore NK cell infiltration and function in PDAC TME hold great value for improving therapeutic outcomes.

## 6 Emerging roles for CD4 T and B cells in PDAC TME

### 6.1 CD4 helper T cells: Th1, Th2, and Th17

CD4 T cells are major players and coordinators of innate and adaptive immune responses and have been increasingly implicated in cancer immunity. Upon functional polarization, they show a broad spectrum of differentiation into defined subsets, including T helper 1 (Th1), Th2, Th17, and Treg (discussed above), implying their functions in tumor immunity are multifaceted and highly dependent on contexts (19–22). Th1 cells have been well-recognized to mediate anti-tumor effects, as they produce effector cytokines IL-2 and IFNγ (19, 20). However, a lower abundance of Th1 cells in PDAC was also implicated in the prolonged survival of patients, although the underlying reasons remain undetermined (20). Th2 cells secrete type 2 cytokines IL-4, IL-5, and IL-13, which mediate macrophage immunosuppressive polarization, fibrotic responses, and angiogenesis in tumors (19, 21). In PDAC patients, Th2 cell frequency has been shown an inverse correlation with overall survival, highly suggesting a tumor-supporting role for these cells (19, 21). Nonetheless, Jacenik et al. reported that Th2 cells suppressed colon and pancreatic tumor growth in mice. Mechanistically, it was associated with Th2 cell-secreted IL-5, which promoted anti-tumorigenic responses of macrophages and eosinophils (96). As the main producer of IL-17 family cytokines, Th17 cells have been shown divergent effects in tumor immunity. He et al. provided data showing that elevated Th17 cells and their cytokines IL-17 and IL-22 were associated with tumor invasiveness, metastasis, and poor survival of PDAC patients (97). In line with the study in humans, McAllister et al. reported a remarked reduction in tumor progression in a mouse model of PDAC, of which Th17 cells were depleted (98). In the study, overexpression of IL-17A cytokine in the pancreas significantly accelerated PanIN initiation and progression in mouse models, suggesting a tumor-promoting role for IL-17 signaling albeit the molecular mechanisms required further investigation (98). Interestingly, there were also studies to report the potential anti-tumor effects of Th17 cells, as increased Th17 cell infiltration was positively correlated with tumor control and survival of PDAC mouse models (22). Therefore, the paradoxical effects among Th1, Th2, and Th17 cells in tumor immunity may highly rely on contexts including PDAC TME status, which requires further characterization in order to use their anti-tumor immunity whereas reverse the tumor-promoting role for PDAC therapy.

### 6.2 B cells

B cells are highly infiltrated in PDAC, and their roles in cancer immunity have been the subject of increasing research (23, 99). By determining PanIN and PDAC lesions from both humans and mouse models, Pylayeva-Gupta et al. observed the prominent presence of B cells and that orthotopic PDAC growth was significantly slowed in B cell-deficient mice. Further analysis identified the contribution of IL-35-producing CD1dhiCD5+ B cells to PDAC progression in mice and that these cells were recruited through CXCL13 (100). A regulatory B cell population has been well-documented in PDAC, which, except for IL-35, was also characterized by the expression of IL-10 and PD-L1 (99). It thus explained the capabilities of the B cells to suppress anti-tumor immunity and promote PDAC. Besides, B cells have been implicated in other mechanisms contributing to PDAC progression. They have been found to play a role in programming tumor-supporting FCγR+ TAMs and to be functionally associated with hypoxia in PDAC (101, 102). Collectively, these studies highlighted a tumor-promoting role for B cells in PDAC albeit through various mechanisms. More recently, ectopic lymphoid aggregates, namely tertiary lymph structures (TLS), have been observed in many solid cancers including PDAC. Composed of organized B cells and T cells, TLS presence has been positively associated with immunotherapy efficacy and favorable survival of PDAC patients (103, 104). Underlying mechanisms most likely attributed to TLS functioning as tumor immunity hub readily available in TME (103, 104). In addition, it is postulated that the presence of sparse or organized B cells within tumors may play divergent roles in tumor immunity.

## 7 Cancer-associated fibroblasts in the immunosuppressive TME of PDAC

Cancer-associated fibroblasts (CAFs) represent the most abundant cell type in the TME of PDAC, in which they constitute up to 80% of all cells. CAFs behave with remarkable desmoplastic reaction, a typical feature of the PDAC TME that is largely involved in ECM deposition and vessel remodeling. CAFs are very heterogeneous populations in terms of cellular origin and function (24). Studies have shown that CAFs can derive from pancreatic stellate cells (PSCs), tissue-resident fibroblasts, adipocytes, pericytes, bone marrow-derived progenitors, and endothelial cells (24). PSCs have long been considered as the primary source of CAFs in PDAC, however, cell lineage tracing study targeting Fabp4+ PSCs showed them contributing to a numerically minor CAF subpopulation (24, 105). This suggests that multiple cellular origins contribute to the heterogeneity of CAFs in PDAC (24, 105). The extent to which each potential cellular origin contributes to the diverse population of CAFs in PDAC is still largely unknown. Additionally, the relationship between the different cellular origins and the phenotypic, spatial, and functional heterogeneity of CAFs in PDAC remains unclear.

In the context of PDAC, three subsets of CAFs have been widely appreciated from early efforts by scRNA sequencing analysis of tumors from mouse models and human patients. A myofibroblast-like subset of CAFs (myoCAF) was evidenced by upregulating expression of αSMA and ECM, meanwhile inflammatory CAFs (iCAF) expressed cytokines and chemokines such as IL-6 and CXCL12. Spatially, myoCAFs were located close to the neoplastic cells whereas iCAFs distributed distantly from the tumor cells, likely indicating the distinct modes of CAF-tumor interactions (106–108). In addition to myoCAFs and iCAFs, a distinct CAF population expressing high levels of antigen presentation molecules such as MHC-II molecule and CD74 has been characterized (termed antigen-presenting CAFs, or apCAF). Interestingly, these cells lacked costimulatory molecules, suggesting their inability of mounting a functional immune response (107, 109). Recently, a subset of CAFs expressing leucine-rich-repeat-containing protein 15 (LRRC15) was identified in PDAC, but not in the healthy pancreas, in both mice and humans. LRRC15 marked a myofibroblast population of CAFs that were dependent on TGFβ, although its function in CAFs were unknown. These cells were shown to promote tumor growth and limit anti-tumor immunity and responsiveness to immune checkpoint blockade (110, 111).

CAFs contribute to the immunosuppressive TME in PDAC in various manners. CAFs have been reported to promote the differentiation and/or recruitment of MDSCs in the TME by secreting IL-6, GM-CSF, and CCL2 (24, 106). A more recent study has shown that CAFs secreted CSF-1 to drive p21-mediated TAM proliferation and immunosuppressive phenotypes, which promoted PDAC progression (112). Moreover, CAFs impaired anti-tumor T cell immunity, through CXCL12-mediated T cell exclusion and/or TGFβ-mediated T cell functional suppression (24, 88, 89). Finally, costimulatory-deficient apCAFs presented antigens to T cells but were unable to activate them. ApCAFs thus prevented T cells from being activated by professional antigen-presenting cells. More recently, apCAFs were shown to have an immunoregulatory function since they directly induced Treg differentiation from naïve CD4 T cells in an antigen-specific manner (107, 109). In summary, the fundamental investigation of CAF origin, phenotypic and functional heterogeneity, and how they contribute to the immunosuppressive TME in PDAC will generate instrumental knowledge for targeting them.

## 8 Targeting the immunosuppressive TME to improve PDAC therapy

Immunotherapies with immune checkpoint inhibitors (ICI) have revolutionized the treatment of several cancers. However, this new treatment, particularly monotherapy, seems not to be entirely effective for PDAC, except for the 1% of patients harboring high microsatellite instability in tumors. Reasons that contribute to the low efficacy of ICI therapy for PDAC are multiple, with the overarching TME representing the most notorious one (2–5). In this regard, TME-targeted strategies have long been investigated to improve PDAC therapy, among which novel examples will be highlighted in the section (**Table 1**).

**Table 1:** Selected clinical trials targeting TME for pancreatic cancer therapy.

Target	Class	Agent	Combination partners	Enrolled patients (n)	Response of combined treatment arm	Reported biological responses	Population	Phase	Trial status	Clinical trial
CSF1R	CSF1R inhibitor (mAb)	Cabiralizumab	FOLFIRINOX, Gemcitabine/Nab-Paclitaxel, anti-PD-1 (nivolumab)	206	NR	NR	Advanced PDAC	II	Complete: 06/01/2023	NCT03336216
CD40	CD40 agonist (mAb)	CP-870,893	Gemcitabine	21	ORR 40%; SDR 53%	Inflammation cytokines (up); B cells (down)	Advanced PDAC	I	Complete: 01/2011	NCT00711191
	CD40 agonist (mAb)	APX005M	Gemcitabine/Nab-Paclitaxel, anti-PD-1 (Nivolumab)	129	ORR 31%; SDR 69%	Intratumoral CD4T cells (up); circulating differentiated CD4T cells and antigen-presenting cells (up)	Metastatic PDAC	I/II	Complete: 02/25/2022	NCT03214250
CCR2	CCR2 antagonist (small molecule)	PF-04136309	FOLFIRINOX	44	ORR 49%; SDR 14%	Peripheral CCR2+ monocytes (down), TAMs (down); Tumoral Tregs (down), CD4T and CD8T cells (up); tumoral IL12a and TNFa mRNA (up); IL10, TGFβ, IL13 mRNA (down)	PDAC	Ib	Complete: 09/2016	NCT01413022
	CCR2 antagonist (small molecule)	PF-04136309	Gemcitabine/Nab-Paclitaxel	21	NR	Peripheral CD14+CCR2+ inflammatory monocytes (down)	Metastatic PDAC	Ib/II	Complete: 10/10/2017	NCT02732938
	CCR2 antagonist (small molecule)	CCX872-B	FOLFIRINOX	54	ORR 30-37%; DCR 78%	Peripheral CCR2+ monocytes (down); tumoral MDSC (down), TAMs (down), Tregs (down); CD4T and CD8T cells (up)	PDAC	Ib	Complete: 05/06/2020	NCT02345408
CCR2/5	Dual antagonist (small molecule)	BMS-813160	Gemcitabine/Nab-Paclitaxel, anti-PD-1 (Nivolumab)	40	NR	NR	Advanced PDAC	I/II	Estimated: 10/14/2024	NCT03496662
	Dual antagonist (small molecule)	BMS-813160	FOLFIRINOX, anti-PD-1 (Nivolumab)	332	NR	NR	including advanced PDAC	Ib/II	Complete: 06/14/2023	NCT03184870
CXCR2	CXCR2 antagonist (small molecule)	AZD5069	anti-PD-L1 (Durvalumab)	23	ORR 5.6%; SDR 11% at 6mos; 5.6% at 12mos	NR	Metastatic PDAC	Ib/II	Complete: 07/09/2018	NCT02583477
CXCR1/2	CXCR1/2 inhibitor (small molecule)	SX-682	anti-PD-1 (nivolumab)	20	NR	NR	PDAC	I	Estimated: 12/31/2024	NCT04477343
CXCR4	AMD3100 (small molecule)	Plerixafor	anti-PD-1 (Cemiplimab)	25	NR	Intratumor effector T cells (up); macrophages and neutrophils (up)	Metastatic pnacreatic cancer	II	Complete: 0519/2023	NCT04177810
	BL-8040 (small molecule)	Motixafortide	anti-PD-1 (Pembrolizumab), fluorouracil (5-FU) and leucovorin (LV)	80	ORR 32%; DCR 77%	Tumoral CD8+ effector T cells (up); MDSCs (down); circulating Tregs (down)	Metastatic pnacreatic cancer	II	Complete: 09/06/2022	NCT02826486
TGFβ	Anti-TGFβ (mAb)	NIS793	Gemcitabine/Nab-Paclitaxel, anti-PD-1 (Spartalizumab)	151	NR	NR	Metastatic PDAC	II	Estimated: 11/30/2023	NCT04390763
	TGFβRI inhibitor (small molecule)	Galunisertib	anti-PD-L1 (Durvalumab)	37	ORR 3.1%; DCR 25%	NR	Metastatic pnacreatic cancer	I	Complete: 04/17/2019	NCT02734160
IL-10	Pegylated IL-10	Pegilodecakin	Folinic acid, fluorouracil and oxaliplatin (FOLFOX)	567	ORR 4.6%	IL-18, IFN-γ, and granzyme B (up); TGFβ (down)	Pancreatic cancer	III	Complete: 03/05/2020	NCT02923921
Vaccine	Allogeneic GM-CSF-secreting cells	GVAX	CRS-207, Cy, anti-PD-1 (Nivolumab), anti-CTLA4 (Ipilimumab)	61	ORR 4%	CD8T cell (up); CD68+ myeloid cells (down)	PDAC	II	Estimated: 08/01/2023	NCT03190265

mAb, monoclonal antibody; CSF1R, colony-stimulating factor-1 receptor; FOLFIRINOX, leucovorin calcium (folinic acid), fluorouracil, irinotecan hydrochloride, and oxaliplatin; NR, not reported; PDAC, pancreatic ductal adenocarcinoma; ORR, objective response rate; SDR, stable disease rate; up, increase in analysis; down, decrease in analysis; TAM, tumor-associated macrophage; Treg, regulatory T cell; DCR, disease control rate; MDSC, myeloid-derived suppressor cell; PD1, programmed death protein 1; PD-L1, programmed cell death ligand 1; mos, months; Estimated, estimated complete date; TGFβRI, TGFβ type I receptor; GVAX, GM-CSF-secreting pancreatic cancer cell lines; CRS-207, live-attenuated listeria-encoding human mesothelin vaccine; Cy, cyclophosphamide; CTLA4, cytotoxic T-lymphocyte-associated protein 4.

### 8.1 Targeting the immunosuppression

CSF1/CSF1R pathway plays a crucial role in TAM recruitment, maintenance, and proliferation, which can be prevented either with monoclonal antibodies to block CSF1R dimerization or with small molecule inhibitors to impair CSF1R-mediated signal transduction (44, 45, 113, 114). CSF1R inhibition has been shown to reduce CD206hi TAMs in PDAC, thereby leading to M1-like macrophage polarization, increased T cell infiltration, and reduced tumor growth (113, 114). Importantly, CSF1R inhibition improved radiotherapy, anti-PD1 and anti-CTLA4 immunotherapies, and gemcitabine chemotherapy in preclinical mouse models of PDAC (113, 114). However, a cautious approach must be taken for future clinical applications due to the potential compensatory effect of TAM depletion, which may lead to the emergence of immunosuppressive G-MDSCs (66, 115).

CCL2/CCR2 axis is highly used for PDAC to mobilize and recruit inflammatory monocytes, which further differentiate into TAMs in TME (44, 45, 116, 117). In mice, pharmacologically blocking CCL2/CCR2 axis through an anti-CCL2 neutralizing antibody or CCR2 inhibitor resulted in reduced CCR2+ monocytes and TAMs in primary PDAC and pre-metastatic liver, which consequently contributed to improved anti-tumor immunity, reduced tumor growth, and decreased metastasis (116, 117). Notably, discrepancies have been observed in murine models when comparing the effects of pharmacological blockade of CCL2/CCR2 axis to those of germline genetic ablation of CCR2 in attenuating PDAC progression (53). Such findings underscore the necessity for more meticulous and comprehensive consideration when utilizing preclinical animal models in future research. In addition, CCL2-mediated recruitment of monocytes has been a critical mechanism for PDAC to resist radiotherapy, given that blocking CCL2/CCR2 axis improved ablative radiotherapy in mouse models of PDAC (116). Clinically, phase I trials NCT01413022 (CCR2 antagonist PF-04136309 + FOLFIRINOX) and NCT02345408 (CCR2 antagonist CCX872 + FOLFIRINOX) have seen objective responses for the PDAC patients treated with the combinations (118, 119).

Another strategy to target TAMs in PDAC involves the application of CD40 agonists to activate their anti-tumor responses (14, 87, 93, 120). CD40, a member of TNF superfamily, is broadly expressed by immune cells, including monocytes, macrophages, and DCs, and is crucial for their activation, antigen presentation, and other immune responses (93, 120). In mouse models of PDAC, treatment with agonistic CD40 antibodies reprogramed TAMs toward anti-tumor phenotypes. It was evidenced by the upregulation of MHC-II and CD86, and elevated production of pro-inflammatory cytokines IL-12, TNFα, and IFNγ (93, 120). Further, combined treatment with CD40 agonists and gemcitabine/nab-paclitaxel improved TAM responses and anti-tumor T-cell clonal expansion, consequently facilitating PDAC control in mouse models (87, 93, 120). Moreover, triple therapy with T-cell inducting vaccine, PD-1 blockade, and CD40 agonist significantly promoted anti-tumor T cell immunity, marked by elevated infiltration of IFNγ-, TNFα-, and granzyme B-secreting effector T cells (121). As a result, triple therapy further improved tumor control and prolonged mouse survival. Of note, macrophage depletion markedly compromised the anti-tumor effect of CD40 agonist, suggesting the significance of macrophages in the application of this therapy (121). In patients with PDAC, combined treatment with CD40 agonist (CP-870,893) and gemcitabine led to a reduction in tumor burden in phase I study (NCT00711191) (120). However, the phase II clinical trial (NCT03214250) for metastatic PDAC patients treated with the combination of CD40 agonist (Sotigalimab), gemcitabine/nab-paclitaxel, and PD-1 blockade (Nivolumab) did not show improvements in 1-year overall survival rates (122). Therefore, future studies to identify predictive biomarkers of response will be required to achieve higher efficiency.

TANs are abundant in PDAC and targeting them has been a subject of extensive research. TAN depletion with a small molecule inhibitor of CXCR2 led to a remarked reduction in PDAC progression and metastasis in mice, which was associated with improved T cell infiltration. In line with this, CXCR2 inhibition further synergized with anti-PD1 and/or FOLFIRINOX therapies (66, 67). However, PDAC patients treated with combined CXCR2 inhibitor (AZD5069) and anti-PD-L1 (Durvalumab) in a phase Ib/II clinical trial (NCT02583477) demonstrated limited efficacy, which warranted future studies. It has been shown that CXCR2 inhibition resulted in compensatory emergence of CCR2+ myeloid cells in mouse PDAC, which in turn remarkably compromised the effect of CXCR2 inhibition (66). Further, combined inhibition of CXCR2 and CCR2 successfully disrupted the recruitment of immunosuppressive myeloid cells in mouse PDAC and consequently improved chemotherapy responses (66). It suggests an important point to be considered in future clinical trials regarding therapies through myeloid cell depletion.

Different strategies to directly target Tregs have been investigated. One of the earliest studies was the incorporation of low-dose cyclophosphamide in different treatment regimens to target Tregs (123, 124). Studies showed that Tregs had higher susceptibility to the toxic effects of cyclophosphamide due to their low levels of intracellular ATP (Adenosine triphosphate) and glutathione, thus were selectively eliminated (123, 124). In combination with the allogeneic PDAC vaccine (GVAX, granulocyte macrophage colony-stimulating factor–secreting pancreatic cancer cell lines), cyclophosphamide has been shown to augment immune responses in PDAC patients (125, 126). Additionally, CTLA-4, neuropilin-1, and CCL5/CCR5 have been explored as targets for intratumoral Tregs (2, 4, 5). However, it is especially worth noting that a recent study has shown an acceleration of tumorigenesis in the context of Foxp3+ Treg cell-genetic depletion in a mouse model of PDAC, which mechanistically attributed to compensatory infiltration of myeloid cells, in particular TAMs (83). In this regard, chemotherapies that can delete Tregs, such as low-dose gemcitabine (127), could unintendedly contribute to pro-tumor consequences in PDAC patients. Thus, these studies imply that therapeutic strategies aimed at immunosuppressive cell modulation rather than depletion could hold more potential to benefit PDAC outcomes.

### 8.2 Targeting cancer-associated fibroblasts

Targeting cancer-associated fibroblasts (CAFs) to treat cancer was initially evaluated with inhibitors of fibroblast-activation protein (FAP), a type-II transmembrane serine protease highly expressed by fibroblasts. In mice with subcutaneous PDAC, FAP inhibitor (UAMC-1110) did not show any meaningful efficacy as a single agent (128). Similarly, in patients with metastatic PDAC, combined treatment with FAP inhibitor (Talabostat) and gemcitabine demonstrated very limited efficacy over historical gemcitabine monotherapy in a phase II clinical trial (129). Given the lack of success in targeting FAP, subsequent studies have been investigated to deplete active CAFs. Studies have shown that genetic depletion of aSMA-expressing CAFs (myoCAF) in mouse models of PDAC promoted tumor progression, suggesting a tumor-suppressing function of these cells (130). Interestingly, a recent study by Krishnamurty et al. reported that depletion of LRRC15+ myoCAFs slowed tumor growth in mouse models of subcutaneous PDAC (111). Moreover, LRRC15+ myoCAF depletion in combination with anti-PDL1 led to a significantly improved anti-tumor effect (111). According to these findings, the study instead noted a tumor-supporting role for LRRC15+ myoCAFs in PDAC (111). Notably, the paradoxical results of targeting myoCAFs from the abovementioned studies warranted a comprehensive understanding of CAF heterogeneity in PDAC therapy. In PDAC, ECM is primarily secreted by CAFs and highly deposited in the TME (24). Targeting ECM, such as modulating sonic hedgehog signaling, MMP activity, or hyaluronan deposition, has also been studied. Unfortunately, early clinical trials in PDAC patients did not yield satisfactory therapeutic efficacy with these strategies (24). Another strategy for targeting CAFs is to block CAF-mediated immunosuppression. For example, disrupting CXCL12-CXCR4 signaling by AMD3100, a small molecule inhibitor of CXCR4, demonstrated a synergistic anti-tumor activity in combination with anti-PD-1/PD-L1 therapy in mouse models of PDAC (84, 89). The combination therapy of CXCR4 inhibition (ADM3100) and anti-PD1 (Cemiplimab) is now being studied in a phase II clinical trial (NCT04177810) for patients with metastatic pancreatic cancer (131). CXCR4 inhibition has also been shown to result in the infiltration of additional myeloid cells into tumors, suggesting a potential mechanism of resistance against CXCR4-targeted therapies (131). Together, these findings generally raise a perspective that future strategies should aim at modulating the TME instead of targeted depletion.

## 9 Concluding remarks

Over the past years, increasing knowledge has been made in understanding the complex TME of PDAC and its significance on disease biology and treatment outcomes. Despite its heterogeneity and complex interplay among various cellular components, the PDAC TME consistently exhibits immunosuppressive characteristics, which strongly influence tumor progression, metastasis, as well as responses to therapies. Other research topics that were not covered due to the scope of this review, such as cancer metabolism, vessel remodeling, and cancer vaccines, can also be promisingly targeted for therapeutics. Overall, it can be expected that conceptual advances that understand the overarching TME of PDAC toward a comprehensive overview could help to develop new therapeutic strategies aimed at targeting multiple mechanisms with synergistic effects.

## Author contributions

JG: Conceptualization, Writing – original draft, Writing – review & editing. SW: Conceptualization, Writing – review & editing. QG: Conceptualization, Writing – review & editing.

## References

[1] Siegel RL Miller KD Wagle NS Jemal A. Cancer statistics, 2023. CA Cancer J Clin (2023) 73:17–48. doi: 10.3322/caac.21763 36633525

[2] Halbrook CJ Lyssiotis CA di Magliano MP Maitra A. Pancreatic cancer: Advances and challenges. Cell (2023) 186:1729–54. doi: 10.1016/j.cell.2023.02.014 PMC10182830 37059070

[3] Springfeld C Ferrone CR Katz MH Philip PA Hong TS Hackert T. Neoadjuvant therapy for pancreatic cancer. Nat Rev Clin Oncol (2023) 20:318–37. doi: 10.1038/s41571-023-00746-1 36932224

[4] Sherman MH Beatty GL. Tumor microenvironment in pancreatic cancer pathogenesis and therapeutic resistance. Annu Rev Pathol: Mech Dis (2023) 18:123–48. doi: 10.1146/annurev-pathmechdis-031621-024600 PMC9877114 36130070

[5] Falcomatà C Bärthel S Schneider G Rad R Schmidt-Supprian M Saur D. Context-specific determinants of the immunosuppressive tumor microenvironment in pancreatic cancer. Cancer Discov (2023) 13:278–97. doi: 10.1158/2159-8290.CD-22-0876 PMC9900325 36622087

[6] Bear AS Vonderheide RH O'Hara MH. Challenges and opportunities for pancreatic cancer immunotherapy. Cancer Cell (2020) 38:788–802. doi: 10.1016/j.ccell.2020.08.004 32946773 PMC7738380

[7] Li Y Xiang S Pan W Wang J Zhan H Liu S. Targeting tumor immunosuppressive microenvironment for pancreatic cancer immunotherapy: Current research and future perspective. Front Oncol (2023) 13:1166860. doi: 10.3389/fonc.2023.1166860 37064113 PMC10090519

[8] Karamitopoulou E. Tumour microenvironment of pancreatic cancer: immune landscape is dictated by molecular and histopathological features. Br J Cancer (2019) 121:5–14. doi: 10.1038/s41416-019-0479-5 31110329 PMC6738327

[9] Wellenstein MD de Visser KE. Cancer-cell-intrinsic mechanisms shaping the tumor immune landscape. Immunity (2018) 48:399–416. doi: 10.1016/j.immuni.2018.03.004 29562192

[10] Xu L Zhu S Lan Y Yan M Jiang Z Zhu J. Revealing the contribution of somatic gene mutations to shaping tumor immune microenvironment. Briefings Bioinf (2023) 23:bbac064. doi: 10.1093/bib/bbac064 35229870

[11] Waddell N Pajic M Patch AM Chang DK Kassahn KS Bailey P. Whole genomes redefine the mutational landscape of pancreatic cancer. Nature (2015) 518:495–501. doi: 10.1038/nature14169 25719666 PMC4523082

[12] Väyrynen SA Zhang J Yuan C Väyrynen JP Dias Costa A Williams H. Composition, spatial characteristics, and prognostic significance of myeloid cell infiltration in pancreatic cancer. Clin Cancer Res (2021) 27:1069–81. doi: 10.1158/1078-0432.CCR-20-3141 PMC8345232 33262135

[13] Tay C Tanaka A Sakaguchi S. Tumor-infiltrating regulatory T cells as targets of cancer immunotherapy. Cancer Cell (2023) 41:450–65. doi: 10.1016/j.ccell.2023.02.014 36917950

[14] Barry ST Gabrilovich DI Sansom OJ Campbell AD Morton JP. Therapeutic targeting of tumour myeloid cells. Nat Rev Cancer (2023) 23:216–37. doi: 10.1038/s41568-022-00546-2 36747021

[15] Tan Z Lei Y Zhang B Shi S Liu J Yu X. Mechanisms of T-cell exhaustion in pancreatic cancer. Cancers (2020) 12:2274. doi: 10.3390/cancers12082274 32823814 PMC7464444

[16] Hegde S Krisnawan VE Herzog BH Zuo C Breden MA Knolhoff BL. Dendritic cell paucity leads to dysfunctional immune surveillance in pancreatic cancer. Cancer Cell (2020) 37:289–307. doi: 10.1016/j.ccell.2020.02.008 32183949 PMC7181337

[17] Lin JH Huffman AP Wattenberg MM Walter DM Carpenter EL Feldser DM. Type 1 conventional dendritic cells are systemically dysregulated early in pancreatic carcinogenesis. J Exp Med (2020) 217:e20190673. doi: 10.1084/jem.20190673 32453421 PMC7398166

[18] Jun E Song AY Choi JW Lee HH Kim MY Ko DH. Progressive impairment of NK cell cytotoxic degranulation is associated with TGF-β1 deregulation and disease progression in pancreatic cancer. Front Immunol (2019) 10:1354. doi: 10.3389/fimmu.2019.01354 31281312 PMC6598013

[19] Tan Z Lei Y Zhang B Shi S Liu J Yu X. Analysis of immune-related signatures related to CD4+ T cell infiltration with gene co-expression network in pancreatic adenocarcinoma. Front Oncol (2021) 11:674897. doi: 10.3389/fonc.2021.674897 34367961 PMC8343184

[20] Wei R Zhang H Cao J Qin D Deng W Li S. Type 1 T helper cell-based molecular subtypes and signature are associated with clinical outcome in pancreatic ductal adenocarcinoma. Front Cell Dev Biol (2022) 10:839893. doi: 10.3389/fcell.2022.839893 35433680 PMC9011157

[21] Piro G Simionato F Carbone C Frizziero M Malleo G Zanini S. A circulating TH2 cytokines profile predicts survival in patients with resectable pancreatic adenocarcinoma. Oncoimmunology (2017) 6:e1322242. doi: 10.1080/2162402X.2017.1322242 28932629 PMC5599089

[22] Gnerlich JL Mitchem JB Weir JS Sankpal NV Kashiwagi H Belt BA. Induction of Th17 cells in the tumor microenvironment improves survival in a murine model of pancreatic cancer. J Immunol (2021) 185:4063–71. doi: 10.4049/jimmunol.0902609 PMC3693576 20805420

[23] Delvecchio FR Goulart MR Fincham REA Bombadieri M Kocher HM. B cells in pancreatic cancer stroma. World J Gastroenterol (2022) 28:1088. doi: 10.3748/wjg.v28.i11.1088 35431504 PMC8985484

[24] Sherman MH di Magliano MP. Cancer-associated fibroblasts: lessons from pancreatic cancer. Annu Rev Cancer Biol (2023) 7:43–55. doi: 10.1146/annurev-cancerbio-061421-035400

[25] Bailey P Chang DK Nones K Johns AL Patch AM Gingras MC. Genomic analyses identify molecular subtypes of pancreatic cancer. Nature (2016) 531:47–52. doi: 10.1038/nature16965 26909576

[26] Connor AA Gallinger S. Pancreatic cancer evolution and heterogeneity: integrating omics and clinical data. Nat Rev Cancer (2022) 22:131–42. doi: 10.1038/s41568-021-00418-1 34789870

[27] Fang YT Yang WW Niu YR Sun YK. Recent advances in targeted therapy for pancreatic adenocarcinoma. World J Gastrointest Oncol (2023) 15:571. doi: 10.4251/wjgo.v15.i4.571 37123059 PMC10134207

[28] Dias Carvalho P Guimaraes CF Cardoso AP Mendonca S Costa AM Oliveira MJ. KRAS oncogenic signaling extends beyond cancer cells to orchestrate the microenvironment. Cancer Res (2018) 78:7–14. doi: 10.1158/0008-5472.CAN-17-2084 29263151

[29] Ischenko I D’Amico S Rao M Li J Hayman MJ Powers S. KRAS drives immune evasion in a genetic model of pancreatic cancer. Nat Commun (2021) 12:1482. doi: 10.1038/s41467-021-21736-w 33674596 PMC7935870

[30] Pylayeva-Gupta Y Lee KE Hajdu CH Miller G Bar-Sagi D. Oncogenic Kras-induced GM-CSF production promotes the development of pancreatic neoplasia. Cancer Cell (2012) 21:836–47. doi: 10.1016/j.ccr.2012.04.024 PMC3721510 22698407

[31] Bayne LJ Beatty GL Jhala N Clark CE Rhim AD Stanger BZ. Tumor-derived granulocyte-macrophage colony-stimulating factor regulates myeloid inflammation and T cell immunity in pancreatic cancer. Cancer Cell (2012) 21:822–35. doi: 10.1016/j.ccr.2012.04.025 PMC3575028 22698406

[32] Hashimoto S Furukawa S Hashimoto A Tsutaho A Fukao A Sakamura Y. ARF6 and AMAP1 are major targets of KRAS and TP53 mutations to promote invasion, PD-L1 dynamics, and immune evasion of pancreatic cancer. Proc Natl Acad Sci (2019) 116:17450–9. doi: 10.1073/pnas.1901765116 PMC6717289 31399545

[33] Alausa A Lawal KA Babatunde OA Obiwulu ENO Oladokun OC Fadahunsi OS. Overcoming immunotherapeutic resistance in PDAC: SIRPα-CD47 blockade. Pharmacol Res (2022) 181:106264. doi: 10.1016/j.phrs.2022.106264 35597384

[34] Yamamoto K Venida A Yano J Biancur DE Kakiuchi M Gupta S. Autophagy promotes immune evasion of pancreatic cancer by degrading MHC-I. Nature (2020) 581:100–5. doi: 10.1038/s41586-020-2229-5 PMC7296553 32376951

[35] Yamamoto K Venida A Perera RM Kimmelman AC. Selective autophagy of MHC-I promotes immune evasion of pancreatic cancer. Autophagy (2020) 16:1524–5. doi: 10.1080/15548627.2020.1769973 PMC7469632 32459143

[36] Wirth M Mahboobi S Krämer OH Schneider G. Concepts to target MYC in pancreatic cancer. Mol Cancer Ther (2016) 15:1792–8. doi: 10.1158/1535-7163.MCT-16-0050 27406986

[37] Hessmann E Schneider G Ellenrieder V Siveke JT. MYC in pancreatic cancer: novel mechanistic insights and their translation into therapeutic strategies. Oncogene (2016) 35:1609–18. doi: 10.1038/onc.2015.216 26119937

[38] Sodir NM Kortlever RM Barthet VJ Campos T Pellegrinet L Kupczak S. MYC instructs and maintains pancreatic adenocarcinoma phenotype. Cancer Discov (2020) 10:588–607. doi: 10.1158/2159-8290.CD-19-0435 31941709

[39] Maddipati R Norgard RJ Baslan T Rathi KS Zhang A Saeid A. MYC levels regulate metastatic heterogeneity in pancreatic adenocarcinoma. Cancer Discov (2022) 12:542–61. doi: 10.1158/2159-8290.CD-20-1826 PMC8831468 34551968

[40] Muthalagu N Monteverde T Raffo-Iraolagoitia X Wiesheu R Whyte D Hedley A. Repression of the type I interferon pathway underlies MYC-and KRAS-dependent evasion of NK and B cells in pancreatic ductal adenocarcinoma. Cancer Discov (2020) 10:872–87. doi: 10.1158/2159-8290.CD-19-0620 PMC7611248 32200350

[41] Maddalena M Mallel G Nataraj NB Shreberk-Shaked M Hassin O Mukherjee S. TP53 missense mutations in PDAC are associated with enhanced fibrosis and an immunosuppressive microenvironment. Proc Natl Acad Sci (2021) 118:e2025631118. doi: 10.1073/pnas.2025631118 34088837 PMC8201917

[42] Siolas D Vucic E Kurz E Hajdu C Bar-Sagi D. Gain-of-function p53R172H mutation drives accumulation of neutrophils in pancreatic tumors, promoting resistance to immunotherapy. Cell Rep (2021) 36:109578. doi: 10.1016/j.celrep.2021.109578 34433022 PMC8687588

[43] Blagih J Zani F Chakravarty P Hennequart M Pilley S Hobor S. Cancer-specific loss of p53 leads to a modulation of myeloid and T cell responses. Cell Rep (2020) 30:481–96. doi: 10.1016/j.celrep.2019.12.028 PMC6963783 31940491

[44] Yang S Liu Q Liao Q. Tumor-associated macrophages in pancreatic ductal adenocarcinoma: origin, polarization, function, and reprogramming. Front Cell Dev Biol (2021) 8:607209. doi: 10.3389/fcell.2020.607209 33505964 PMC7829544

[45] Pittet MJ Michielin O Migliorini D. Clinical relevance of tumour-associated macrophages. Nat Rev Clin Oncol (2022) 19:402–21. doi: 10.1038/s41571-022-00620-6 35354979

[46] Liou GY Döppler H Necela B Krishna M Crawford HC Raimondo M. Macrophage-secreted cytokines drive pancreatic acinar-to-ductal metaplasia through NF-κB and MMPs. J Cell Biol (2013) 202:563–77. doi: 10.1083/jcb.201301001 PMC3734091 23918941

[47] Liou GY Döppler H Necela B Edenfield B Zhang L Dawson DW. Mutant KRAS–induced expression of ICAM-1 in pancreatic acinar cells causes attraction of macrophages to expedite the formation of precancerous lesions. Cancer Discov (2015) 5:52–63. doi: 10.1158/2159-8290.CD-14-0474 25361845 PMC4293204

[48] Lesina M Kurkowski MU Ludes K Rose-John S Treiber M Klöppel G. Stat3/Socs3 activation by IL-6 transsignaling promotes progression of pancreatic intraepithelial neoplasia and development of pancreatic cancer. Cancer Cell (2011) 19:456–69. doi: 10.1016/j.ccr.2011.03.009 21481788

[49] Al-Ismaeel Q Neal CP Al-Mahmoodi H Almutairi Z Al-Shamarti I Straatman K. ZEB1 and IL-6/11-STAT3 signalling cooperate to define invasive potential of pancreatic cancer cells via differential regulation of the expression of S100 proteins. Br J Cancer (2019) 121:65–75. doi: 10.1038/s41416-019-0483-9 31123345 PMC6738112

[50] Zhang J Song J Tang S Zhao Y Wang L Luo Y. Multi-omics analysis reveals the chemoresistance mechanism of proliferating tissue-resident macrophages in PDAC via metabolic adaptation. Cell Rep (2023) 42:112620. doi: 10.1016/j.celrep.2023.112620 37285267

[51] Griesmann H Drexel C Milosevic N Sipos B Rosendahl J Gress TM. Pharmacological macrophage inhibition decreases metastasis formation in a genetic model of pancreatic cancer. Gut (2017) 66:1278–85. doi: 10.1136/gutjnl-2015-310049 27013602

[52] Gao Q Mo S Han C Liao X Yang C Wang X. Comprehensive analysis of LILR family genes expression and tumour-infiltrating immune cells in early-stage pancreatic ductal adenocarcinoma. IET Syst Biol (2023) 17:39–57. doi: 10.1049/syb2.12058 36748687 PMC10116025

[53] Zhu Y Herndon JM Sojka DK Kim KW Knolhoff BL Zuo C. Tissue-resident macrophages in pancreatic ductal adenocarcinoma originate from embryonic hematopoiesis and promote tumor progression. Immunity (2017) 47:323–38. doi: 10.1016/j.immuni.2017.07.014 PMC5578409 28813661

[54] Molgora M Esaulova E Vermi W Hou J Chen Y Luo J. TREM2 modulation remodels the tumor myeloid landscape enhancing anti-PD-1 immunotherapy. Cell (2020) 182:886–900. doi: 10.1016/j.cell.2020.07.013 32783918 PMC7485282

[55] Binnewies M Pollack JL Rudolph J Dash S Abushawish M Lee T. Targeting TREM2 on tumor-associated macrophages enhances immunotherapy. Cell Rep (2021) 37:109844. doi: 10.1016/j.celrep.2021.109844 34686340

[56] Park MD Reyes-Torres I LeBerichel J Hamon P LaMarche NM Hegde S. TREM2 macrophages drive NK cell paucity and dysfunction in lung cancer. Nat Immunol (2023) 24:792–801. doi: 10.1038/s41590-023-01475-4 37081148 PMC11088947

[57] Colonna M. The biology of TREM receptors. Nat Rev Immunol (2023) 7:1–15. doi: 10.1038/s41577-023-00837-1 PMC9904274 36750615

[58] Hedrick CC Malanchi I. Neutrophils in cancer: heterogeneous and multifaceted. Nat Rev Immunol (2022) 22:173–87. doi: 10.1038/s41577-021-00571-6 34230649

[59] Jiang W Li X Xiang C Zhou W. Neutrophils in pancreatic cancer: potential therapeutic targets. Front Oncol (2022) 12:1025805. doi: 10.3389/fonc.2022.1025805 36324574 PMC9618950

[60] Wang Y Fang T Huang L Wang H Zhang L Wang Z. Neutrophils infiltrating pancreatic ductal adenocarcinoma indicate higher Malignancy and worse prognosis. Biochem Biophys Res Commun (2018) 501:313–9. doi: 10.1016/j.bbrc.2018.05.024 29738769

[61] Xiang ZJ Hu T Wang Y Wang H Xu L Cui N. Neutrophil–lymphocyte ratio (NLR) was associated with prognosis and immunomodulatory in patients with pancreatic ductal adenocarcinoma (PDAC). Biosci Rep (2020) 40:BSR20201190. doi: 10.1042/BSR20201190 32510138 PMC7300287

[62] Yang JJ Hu ZG Shi WX Deng T He SQ Yuan SG. Prognostic significance of neutrophil to lymphocyte ratio in pancreatic cancer: a meta-analysis. World J gastroenterol: WJG (2015) 21:2807. doi: 10.3748/wjg.v21.i9.2807 25759553 PMC4351235

[63] Jiang SH Liu D Hu LP Zhang S Yu Y Sun YW. Modeling of cancer-related body-wide effects identifies LTB4 as a diagnostic biomarker for pancreatic cancer. EBioMedicine (2022) 80:104050. doi: 10.1016/j.ebiom.2022.104050 35561453 PMC9108888

[64] Bianchi A De Castro Silva I Deshpande NU Singh S Mehra S Garrido VT. Cell-autonomous Cxcl1 sustains tolerogenic circuitries and stromal inflammation via neutrophil-derived TNF in pancreatic cancer. Cancer Discov (2023) 13:1428–53. doi: 10.1158/2159-8290.CD-22-1046 PMC10259764 36946782

[65] Nielsen SR Strøbech JE Horton ER Jackstadt R Laitala A Bravo MC. Suppression of tumor-associated neutrophils by lorlatinib attenuates pancreatic cancer growth and improves treatment with immune checkpoint blockade. Nat Commun (2021) 12:3414. doi: 10.1038/s41467-021-23731-7 34099731 PMC8184753

[66] Nywening TM Belt BA Cullinan DR Panni RZ Han BJ Sanford DE. Targeting both tumour-associated CXCR2+ neutrophils and CCR2+ macrophages disrupts myeloid recruitment and improves chemotherapeutic responses in pancreatic ductal adenocarcinoma. Gut (2018) 67:1112–23. doi: 10.1136/gutjnl-2017-313738 PMC5969359 29196437

[67] Steele CW Karim SA Leach JD Bailey P Upstill-Goddard R Rishi L. CXCR2 inhibition profoundly suppresses metastases and augments immunotherapy in pancreatic ductal adenocarcinoma. Cancer Cell (2016) 29:832–45. doi: 10.1016/j.ccell.2016.04.014 PMC4912354 27265504

[68] Wang X Hu LP Qin WT Yang Q Chen DY Li Q. Identification of a subset of immunosuppressive P2RX1-negative neutrophils in pancreatic cancer liver metastasis. Nat Commun (2021) 12:174. doi: 10.1038/s41467-020-20447-y 33420030 PMC7794439

[69] Gaida MM Steffen TG Günther F Tschaharganeh DF Felix K Bergmann F. Polymorphonuclear neutrophils promote dyshesion of tumor cells and elastase-mediated degradation of E-cadherin in pancreatic tumors. Eur J Immunol (2012) 42:3369–80. doi: 10.1002/eji.201242628 23001948

[70] Canè S Barouni RM Fabbi M Cuozzo J Fracasso G Adamo A. Neutralization of NET-associated human ARG1 enhances cancer immunotherapy. Sci Trans Med (2023) 15:eabq6221. doi: 10.1126/scitranslmed.abq6221 36921034

[71] Jin W Yin H Li H Yu XJ Xu HX Liu L. Neutrophil extracellular DNA traps promote pancreatic cancer cells migration and invasion by activating EGFR/ERK pathway. J Cell Mol Med (2021) 25:5443–56. doi: 10.1111/jcmm.16555 PMC8184670 33955688

[72] Miller-Ocuin JL Liang X Boone BA Doerfler WR Singhi AD Tang D. DNA released from neutrophil extracellular traps (NETs) activates pancreatic stellate cells and enhances pancreatic tumor growth. Oncoimmunology (2019) 8:e1605822. doi: 10.1080/2162402X.2019.1605822 31428515 PMC6685506

[73] Fridlender ZG Sun J Kim S Kapoor V Cheng G Ling L. Polarization of tumor-associated neutrophil phenotype by TGF-β:”N1” versus “N2” TAN. Cancer Cell (2009) 16:183–94. doi: 10.1016/j.ccr.2009.06.017 PMC2754404 19732719

[74] Gungabeesoon J Gort-Freitas NA Kiss M Bolli E Messemaker M Siwicki M. A neutrophil response linked to tumor control in immunotherapy. Cell (2023) 186:1448–64. doi: 10.1016/j.cell.2023.02.032 PMC10132778 37001504

[75] Hirschhorn D Budhu S Kraehenbuehl L Gigoux M Schröder D Chow A. T cell immunotherapies engage neutrophils to eliminate tumor antigen escape variants. Cell (2023) 186:1432–47. doi: 10.1016/j.cell.2023.03.007 PMC10994488 37001503

[76] Karakhanova S Link J Heinrich M Shevchenko I Yang Y Hassenpflug M. Characterization of myeloid leukocytes and soluble mediators in pancreatic cancer: importance of myeloid-derived suppressor cells. Oncoimmunology (2015) 4:e998519. doi: 10.1080/2162402X.2014.998519 26137414 PMC4485765

[77] Diaz-Montero CM Salem ML Nishimura MI Garrett-Mayer E Cole DJ Montero AJ. Increased circulating myeloid-derived suppressor cells correlate with clinical cancer stage, metastatic tumor burden, and doxorubicin–cyclophosphamide chemotherapy. Cancer Immunol Immunother (2009) 58:49–59. doi: 10.1007/s00262-008-0523-4 18446337 PMC3401888

[78] Veglia F Sanseviero E Gabrilovich DI. Myeloid-derived suppressor cells in the era of increasing myeloid cell diversity. Nat Rev Immunol (2021) 21:485–98. doi: 10.1038/s41577-020-00490-y PMC7849958 33526920

[79] Siret C Collignon A Silvy F Robert S Cheyrol T André P. Deciphering the crosstalk between myeloid-derived suppressor cells and regulatory T cells in pancreatic ductal adenocarcinoma. Front Immunol (2020) 10:3070. doi: 10.3389/fimmu.2019.03070 32038621 PMC6987391

[80] Pergamo M Miller G. Myeloid-derived suppressor cells and their role in pancreatic cancer. Cancer Gene Ther (2017) 24:100–5. doi: 10.1038/cgt.2016.65 27910857

[81] Togashi Y Shitara K Nishikawa H. Regulatory T cells in cancer immunosuppression—implications for anticancer therapy. Nat Rev Clin Oncol (2019) 16:356–71. doi: 10.1038/s41571-019-0175-7 30705439

[82] Liu L Zhao G Wu W Rong Y Jin D Wang D. Low intratumoral regulatory T cells and high peritumoral CD8+ T cells relate to long-term survival in patients with pancreatic ductal adenocarcinoma after pancreatectomy. Cancer Immunol Immunother (2016) 65:73–82. doi: 10.1007/s00262-015-1775-4 26646849 PMC11029368

[83] Zhang Y Lazarus J Steele NG Yan W Lee HJ Nwosu ZC. Regulatory T-cell depletion alters the tumor microenvironment and accelerates pancreatic carcinogenesis. Cancer Discov (2020) 10:422–39. doi: 10.1158/2159-8290.CD-19-0958 PMC7224338 31911451

[84] Ma Y Hwang RF Logsdon CD Ullrich SE. Dynamic mast cell–stromal cell interactions promote growth of pancreatic cancer. Cancer Res (2013) 73:3927–37. doi: 10.1158/0008-5472.CAN-12-4479 PMC3702652 23633481

[85] Chang DZ Ma Y Ji B Wang H Deng D Liu Y. Mast cells in tumor microenvironment promotes the in *vivo* growth of pancreatic ductal adenocarcinoma. Clin Cancer Res (2011) 17:7015–23. doi: 10.1158/1078-0432.CCR-11-0607 PMC4089502 21976550

[86] Longo V Tamma R Brunetti O Pisconti S Argentiero A Silvestris N. Mast cells and angiogenesis in pancreatic ductal adenocarcinoma. Clin Exp Med (2018) 18:319–23. doi: 10.1007/s10238-018-0493-6 29492715

[87] Beatty GL Winograd R Evans RA Long KB Luque SL Lee JW. Exclusion of T cells from pancreatic carcinomas in mice is regulated by Ly6Clow F4/80+ extratumoral macrophages. Gastroenterology (2015) 149:201–10. doi: 10.1053/j.gastro.2015.04.010 PMC4478138 25888329

[88] Principe DR Park A Dorman MJ Kumar S Viswakarma N Rubin J. TGFβ blockade augments PD-1 inhibition to promote T-cell–mediated regression of pancreatic cancer. Mol Cancer Ther (2019) 18:613–20. doi: 10.1158/1535-7163.MCT-18-0850 PMC6397698 30587556

[89] Feig C Jones JO Kraman M Wells RJ Deonarine A Chan DS. Targeting CXCL12 from FAP-expressing carcinoma-associated fibroblasts synergizes with anti–PD-L1 immunotherapy in pancreatic cancer. Proc Natl Acad Sci (2013) 110:20212–7. doi: 10.1073/pnas.1320318110 PMC3864274 24277834

[90] Ene–Obong A Clear AJ Watt J Wang J Fatah R Riches JC. Activated pancreatic stellate cells sequester CD8+ T cells to reduce their infiltration of the juxtatumoral compartment of pancreatic ductal adenocarcinoma. Gastroenterology (2013) 145:1121–32. doi: 10.1053/j.gastro.2013.07.025 PMC3896919 23891972

[91] Yamamoto T Yanagimoto H Satoi S Toyokawa H Yamao J Kim S. Circulating myeloid dendritic cells as prognostic factors in patients with pancreatic cancer who have undergone surgical resection. J Surg Res (2012) 173:299–308. doi: 10.1016/j.jss.2010.09.027 21195425

[92] James CA Baer JM Zou C Panni UY Knolhoff BL Hogg GD. Systemic alterations in type-2 conventional dendritic cells lead to impaired tumor immunity in pancreatic cancer. Cancer Immunol Res (2023) 11:1055–67. doi: 10.1158/2326-6066.CIR-21-0946 PMC10524961 37229629

[93] Lau SP van Montfoort N Kinderman P Lukkes M Klaase L van Nimwegen M. Dendritic cell vaccination and CD40-agonist combination therapy licenses T cell-dependent antitumor immunity in a pancreatic carcinoma murine model. J immunother Cancer (2020) 8:e000772. doi: 10.1136/jitc-2020-000772 32690771 PMC7373331

[94] Lim SA Kim J Jeon S Shin MH Kwon J Kim TJ. Defective localization with impaired tumor cytotoxicity contributes to the immune escape of NK cells in pancreatic cancer patients. Front Immunol (2019) 10:496. doi: 10.3389/fimmu.2019.00496 31024520 PMC6465515

[95] Liu B Zhu X Kong L Wang M Spanoudis C Chaturvedi P. Bifunctional TGF-β trap/IL-15 protein complex elicits potent NK cell and CD8+ T cell immunity against solid tumors. Mol Ther (2021) 29:2949–62. doi: 10.1016/j.ymthe.2021.06.001 PMC8531151 34091051

[96] Jacenik D Karagiannidis I Beswick EJ. Th2 cells inhibit growth of colon and pancreas cancers by promoting anti-tumorigenic responses from macrophages and eosinophils. Br J Cancer (2023) 128:387–97. doi: 10.1038/s41416-022-02056-2 PMC9902541 36376448

[97] He S Fei M Wu Y Zheng D Wan D Wang L. Distribution and clinical significance of Th17 cells in the tumor microenvironment and peripheral blood of pancreatic cancer patients. Int J Mol Sci (2011) 12:7424–37. doi: 10.3390/ijms12117424 PMC3233413 22174607

[98] McAllister F Bailey JM Alsina J Nirschl CJ Sharma R Fan H. Oncogenic Kras activates a hematopoietic-to-epithelial IL-17 signaling axis in preinvasive pancreatic neoplasia. Cancer Cell (2014) 25:621–37. doi: 10.1016/j.ccr.2014.03.014 PMC4072043 24823639

[99] Senturk ZN Akdag I Deniz B Sayi-Yazgan A. Pancreatic cancer: Emerging field of regulatory B-cell-targeted immunotherapies. Front Immunol (2023) 14:1152551. doi: 10.3389/fimmu.2023.1152551 37033931 PMC10076755

[100] Pylayeva-Gupta Y Das S Handler JS Hajdu CH Coffre M Koralov SB. IL35-producing B cells promote the development of pancreatic neoplasia. Cancer Discovery (2016) 6:247–55. doi: 10.1158/2159-8290.CD-15-0843 PMC5709038 26715643

[101] Gunderson AJ Kaneda MM Tsujikawa T Nguyen AV Affara NI Ruffell B. Bruton tyrosine kinase–dependent immune cell cross-talk drives pancreas cancer. Cancer Discovery (2016) 6:270–85. doi: 10.1158/2159-8290.CD-15-0827 PMC4783268 26715645

[102] Lee KE Spata M Bayne LJ Buza EL Durham AC Allman D. Hif1a deletion reveals pro-neoplastic function of B cells in pancreatic neoplasia. Cancer Discovery (2016) 6:256–69. doi: 10.1158/2159-8290.CD-15-0822 PMC4783189 26715642

[103] Castino GF Cortese N Capretti G Serio S Di Caro G Mineri R. Spatial distribution of B cells predicts prognosis in human pancreatic adenocarcinoma. Oncoimmunology (2016) 54:e1085147. doi: 10.1080/2162402X.2015.1085147 PMC4839336 27141376

[104] Kinker GS Vitiello GAF Diniz AB Cabral-Piccin MP Pereira PHB Carvalho MLR. Mature tertiary lymphoid structures are key niches of tumour-specific immune responses in pancreatic ductal adenocarcinomas. Gut (2023), gutjnl–2022-328697. doi: 10.1136/gutjnl-2022-328697 37230755

[105] Helms EJ Berry MW Chaw RC DuFort CC Sun D Onate MK. Mesenchymal lineage heterogeneity underlies nonredundant functions of pancreatic cancer–associated fibroblasts. Cancer Discovery (2022) 12:484–501. doi: 10.1158/2159-8290.CD-21-0601 34548310 PMC8831457

[106] Öhlund D Handly-Santana A Biffi G Elyada E Almeida AS Ponz-Sarvise M. Distinct populations of inflammatory fibroblasts and myofibroblasts in pancreatic cancer. J Exp Med (2017) 214:579–96. doi: 10.1084/jem.20162024 PMC5339682 28232471

[107] Elyada E Bolisetty M Laise P Flynn WF Courtois ET Burkhart RA. Cross-species single-cell analysis of pancreatic ductal adenocarcinoma reveals antigen-presenting cancer-associated fibroblasts. Cancer Discov (2019) 9:1102–23. doi: 10.1158/2159-8290.CD-19-0094 PMC6727976 31197017

[108] Bernard V Semaan A Huang J San Lucas FA Mulu FC Stephens BM. Single-cell transcriptomics of pancreatic cancer precursors demonstrates epithelial and microenvironmental heterogeneity as an early event in neoplastic progression. Clin Cancer Res (2019) 25:2194–205. doi: 10.1158/1078-0432.CCR-18-1955 PMC6445737 30385653

[109] Huang H Wang Z Zhang Y Pradhan RN Ganguly D Chandra R. Mesothelial cell-derived antigen-presenting cancer-associated fibroblasts induce expansion of regulatory T cells in pancreatic cancer. Cancer Cell (2022) 40:656–73. doi: 10.1016/j.ccell.2022.04.011 PMC9197998 35523176

[110] Dominguez CX Müller S Keerthivasan S Koeppen H Hung J Gierke S. Single-cell RNA sequencing reveals stromal evolution into LRRC15+ myofibroblasts as a determinant of patient response to cancer immunotherapy. Cancer Discov (2020) 10:232–53. doi: 10.1158/2159-8290.CD-19-0644 31699795

[111] Krishnamurty AT Shyer JA Thai M Gandham V Buechler MB Yang YA. LRRC15+ myofibroblasts dictate the stromal setpoint to suppress tumour immunity. Nature (2022) 611:148–54. doi: 10.1038/s41586-022-05272-1 PMC9630141 36171287

[112] Zuo C Baer JM Knolhoff BL Belle JI Liu X Alarcon de la Lastra A. Stromal and therapy-induced macrophage proliferation promotes PDAC progression and susceptibility to innate immunotherapy. J Exp Med (2023) 220:e20212062. doi: 10.1084/jem.20212062 36951731 PMC10072222

[113] Zhu Y Knolhoff BL Meyer MA Nywening TM West BL Luo J. CSF1/CSF1R blockade reprograms tumor-infiltrating macrophages and improves response to T-cell checkpoint immunotherapy in pancreatic cancer models. Cancer Res (2014) 74:5057–69. doi: 10.1158/0008-5472.CAN-13-3723 PMC4182950 25082815

[114] Ho WJ Jaffee EM. Macrophage-targeting by CSF1/1R blockade in pancreatic cancers. Cancer Res (2021) 81:6071–3. doi: 10.1158/0008-5472.CAN-21-3603 PMC9164148 34911778

[115] Loeuillard E Yang J Buckarma E Wang J Liu Y Conboy C. Targeting tumor-associated macrophages and granulocytic myeloid-derived suppressor cells augments PD-1 blockade in cholangiocarcinoma. J Clin Invest (2020) 130:5380–96. doi: 10.1172/JCI137110 PMC7524481 32663198

[116] Kalbasi A Komar C Tooker GM Liu M Lee JW Gladney WL. Tumor-derived CCL2 mediates resistance to radiotherapy in pancreatic ductal adenocarcinoma. Clin Cancer Res (2017) 23:137–48. doi: 10.1158/1078-0432.CCR-16-0870 PMC5195913 27354473

[117] Wang J Saung MT Li K Fu J Fujiwara K Niu N. CCR2/CCR5 inhibitor permits the radiation-induced effector T cell infiltration in pancreatic adenocarcinoma. J Exp Med (2022) 219:e20211631. doi: 10.1084/jem.20211631 35404390 PMC9006312

[118] Nywening TM Wang-Gillam A Sanford DE Belt BA Panni RZ Cusworth BM. Targeting tumour-associated macrophages with CCR2 inhibition in combination with FOLFIRINOX in patients with borderline resectable and locally advanced pancreatic cancer: a single-centre, open-label, dose-finding, non-randomised, phase 1b trial. Lancet Oncol (2016) 17:651–62. doi: 10.1016/S1470-2045(16)00078-4 PMC5407285 27055731

[119] Linehan D Noel MS Hezel AF Wang-Gillam A Eskens F Sleijfer S. Overall survival in a trial of orally administered CCR2 inhibitor CCX872 in locally advanced/metastatic pancreatic cancer: Correlation with blood monocyte counts. ASCO-SITC Clin Immuno-Oncol (2018) 36:92. doi: 10.1200/JCO.2018.36.5_suppl.92

[120] Beatty GL Chiorean EG Fishman MP Saboury B Teitelbaum UR Sun W. CD40 agonists alter tumor stroma and show efficacy against pancreatic carcinoma in mice and humans. Science (2011) 331:1612–6. doi: 10.1126/science.1198443 PMC3406187 21436454

[121] Winograd R Byrne KT Evans RA Odorizzi PM Meyer AR Bajor DL. Induction of T-cell immunity overcomes complete resistance to PD-1 and CTLA-4 blockade and improves survival in pancreatic carcinoma. Cancer Immunol Res (2015) 3:399–411. doi: 10.1158/2326-6066.CIR-14-0215 25678581 PMC4390506

[122] O'Hara MH O'Reilly EM Varadhachary G Wolff RA Wainberg ZA Ko AH. CD40 agonistic monoclonal antibody APX005M (sotigalimab) and chemotherapy, with or without nivolumab, for the treatment of metastatic pancreatic adenocarcinoma: an open-label, multicentre, phase 1b study. Lancet Oncol (2021) 22:118–31. doi: 10.1016/S1470-2045(20)30532-5 33387490

[123] Le DT Jaffee EM. Regulatory T-cell modulation using cyclophosphamide in vaccine approaches: a current perspective. Cancer Res (2012) 72:3439–44. doi: 10.1158/0008-5472.CAN-11-3912 PMC3399042 22761338

[124] Zhao J Cao Y Lei Z Yang Z Zhang B Huang B. Selective depletion of CD4+ CD25+ Foxp3+ regulatory T cells by low-dose cyclophosphamide is explained by reduced intracellular ATP levels. Cancer Res (2010) 70:4850–8. doi: 10.1158/0008-5472.CAN-10-0283 20501849

[125] Laheru D Lutz E Burke J Biedrzycki B Solt S Onners B. Allogeneic granulocyte macrophage colony-stimulating factor–secreting tumor immunotherapy alone or in sequence with cyclophosphamide for metastatic pancreatic cancer: a pilot study of safety, feasibility, and immune activation. Clin Cancer Res (2008) 14:1455–63. doi: 10.1158/1078-0432.CCR-07-0371 PMC2879140 18316569

[126] Lutz ER Wu AA Bigelow E Sharma R Mo G Soares K. Immunotherapy converts nonimmunogenic pancreatic tumors into immunogenic foci of immune regulation. Cancer Immunol Res (2014) 2:616–31. doi: 10.1158/2326-6066.CIR-14-0027 PMC4082460 24942756

[127] Shevchenko I Karakhanova S Soltek S Link J Bayry J Werner J. Low-dose gemcitabine depletes regulatory T cells and improves survival in the orthotopic Panc02 model of pancreatic cancer. Int J Cancer (2013) 133:98–107. doi: 10.1002/ijc.27990 23233419

[128] Young KH McCarty K Friedman D Cottam B Newell P Gough M. Preclinical combination of radiation and fibroblast activation protein inhibition in pancreatic cancer. ASCO Annu Meeting I (2016) 34:e23117. doi: 10.1200/JCO.2016.34.15_suppl.e23117

[129] Nugent FW Cunningham C Barve MA Fisher W Patel H Meiri E. Phase 2 study of talabostat/gemcitabine in Stage IV pancreatic cancer. J Clin Oncol (2007) 25:4616–6. doi: 10.1200/jco.2007.25.18_suppl.4616

[130] Özdemir BC Pentcheva-Hoang T Carstens JL Zheng X Wu CC Simpson TR. Depletion of carcinoma-associated fibroblasts and fibrosis induces immunosuppression and accelerates pancreas cancer with reduced survival. Cancer Cell (2014) 25:719–34. doi: 10.1016/j.ccr.2014.04.005 PMC4180632 24856586

[131] Shin SM Hernandez A Coyne E Zhang Z Mitchell S Durham J. Combination of CXCR4 antagonist and anti-PD1 therapy results in significant mobilization and increased infiltration of myeloid cells into the metastatic liver microenvironment of PDAC. Cancer Res (2023) 83:2270–0. doi: 10.1158/1538-7445.AM2023-2270

